# Obituary

**Published:** 1882-10

**Authors:** 


### OBITUARY.

NOTICE has been received of the death of Dr. Ernst Friederich Gurlt, who died at Berlin on August 13th, in the eighty-eighth year of his age.

Dr. Gurlt obtained his medical degree at Breslau, where he served under the celebrated Otto as anatomical prosector. "De Venarum Deformitatibus" was the subject of his dissertation for the degree. He joined the Berlin Veterinary School in 1825, and taught there for many years. During his long professional career, he labored earnestly in promoting Medical and Veterinary Science. Success crowned his efforts, his services having been recognized not only in Germany but in other countries. The most important among his numerous writings is the "Handbuch der Vergleichenden Anatomie der Haus-Säugethiere," the fine "Hand-Atlas der Anatomie des Pferdes," in folio; the "Lehrbuch der Vergleichenden Physiologie der Haussäugethiere"; the "Anatomie der Hausvögel;" the "Lehrbuch der Pathologische Anatomie," and "Ueber Thierische Missgeburten," the last part of which appeared as late as 1877. He edited, in conjunction with Hertwig, the *Magasin für Thierheilkunde*, the leading professional journal in Germany, from it commencement; and to it he contributed many papers of the highest interest and importance. Gurlt's name will ever be honorably associated with the development of veterinary science in Europe, while it will enhance the reputation of a school which has been prolific in training the greatest masters of this science.

R. P. Lord, M.R.C.V.S., who graduated in 1873, did recently in Baltimore.

E. P. Bowman, V.S., aged 63, died of apoplexy, in Lynn, Mass. He studied under Dr. George H. Dadd, and was a very energetic and worthy member of the profession.

D. C. Riley, V.S., aged 75, was fatally injured by a railroad train at Long Branch. He was a graduate of Dublin University.

